# The genetic architecture of human amygdala volumes and their overlap with common brain disorders

**DOI:** 10.1038/s41398-023-02387-5

**Published:** 2023-03-11

**Authors:** Ya-Nan Ou, Bang-Sheng Wu, Yi-Jun Ge, Yi Zhang, Yu-Chao Jiang, Kevin Kuo, Liu Yang, Lan Tan, Jian-Feng Feng, Wei Cheng, Jin-Tai Yu

**Affiliations:** 1 Department of Neurology, Qingdao Municipal Hospital, Qingdao University, Qingdao, China; 2 Department of Neurology and Institute of Neurology, Huashan Hospital, State Key Laboratory of Medical Neurobiology and MOE Frontiers Center for Brain Science, Shanghai Medical College, Fudan University, National Center for Neurological Disorders, Shanghai, China; 3 Institute of Science and Technology for Brain-Inspired Intelligence, Fudan University, Shanghai, China; 4 Key Laboratory of Computational Neuroscience and Brain-Inspired Intelligence (Fudan University), Ministry of Education, Shanghai, China; 5 Fudan ISTBI—ZJNU Algorithm Centre for Brain-Inspired Intelligence, Zhejiang Normal University, Jinhua, China; 6 MOE Frontiers Center for Brain Science, Fudan University, Shanghai, China; 7 Zhangjiang Fudan International Innovation Center, Shanghai, China

**Keywords:** Neuroscience, Diseases

## Abstract

The amygdala is a crucial interconnecting structure in the brain that performs several regulatory functions, yet its genetic architectures and involvement in brain disorders remain largely unknown. We carried out the first multivariate genome-wide association study (GWAS) of amygdala subfield volumes in 27,866 UK Biobank individuals. The whole amygdala was segmented into nine nuclei groups using Bayesian amygdala segmentation. The post-GWAS analysis allowed us to identify causal genetic variants in phenotypes at the SNP, locus, and gene levels, as well as genetic overlap with brain health-related traits. We further generalized our GWAS in Adolescent Brain Cognitive Development (ABCD) cohort. The multivariate GWAS identified 98 independent significant variants within 32 genomic loci associated (*P* < 5 × 10^−8^) with amygdala volume and its nine nuclei. The univariate GWAS identified significant hits for eight of the ten volumes, tagging 14 independent genomic loci. Overall, 13 of the 14 loci identified in the univariate GWAS were replicated in the multivariate GWAS. The generalization in ABCD cohort supported the GWAS results with the 12q23.2 (RNA gene *RP11-210L7.1*) being discovered. All of these imaging phenotypes are heritable, with heritability ranging from 15% to 27%. Gene-based analyses revealed pathways relating to cell differentiation/development and ion transporter/homeostasis, with the astrocytes found to be significantly enriched. Pleiotropy analyses revealed shared variants with neurological and psychiatric disorders under the conjFDR threshold of 0.05. These findings advance our understanding of the complex genetic architectures of amygdala and their relevance in neurological and psychiatric disorders.

## Introduction

The amygdala is an interconnecting structure located in the temporal lobe that plays a fundamental part in the regulation of memory formation, fear reaction, anxiety behaviors, and encouraging mechanisms [1]. Anatomically, the amygdala is divided into nine nuclei [2], with input-output neuronal circuits carrying out their distinct functions [1, 3]. It has been suggested that amygdala lesions are implicated in multiple neurological and psychiatric disorders, such as Alzheimer’s disease (AD) [4], Lewy body diseases (including Parkinson’s disease [PD] [5] and dementia with Lewy bodies [DLB] [6]), depression [7], and post-traumatic stress disorder (PTSD) [8]. Still, the genetic underpinnings of amygdala nuclei and their relations to brain disorders have remained largely understudied.

Despite previous imaging-genetics studies have attested to the extremely polygenic genetic architecture of the amygdala, with a heritability estimate ranging from 0.34 to 0.83 [9, 10], individual amygdala nuclei are likely to have distinct genetic determinants, given the differences in cytoarchitecture and functional mechanisms. Based on this, recent work suggests that exploiting this distributed nature in multivariate genome-wide association studies (GWAS) approaches can significantly improve the discovery beyond standard GWAS approaches [11]. Further, whether the differential genetic architecture of amygdala nuclei is involved in common brain disorders is unclear since prior GWASs have only focused on the identification of single nucleotide polymorphisms (SNPs) linked to whole amygdala volume [9, 12]. Fortunately, the UK Biobank (UKB) has released the brain imaging data containing nearly 40,000 participants in early 2020, providing an unprecedented number of imaging-genetics resources and opportunities for an in-depth examination. The adoption of multivariate and univariate GWAS and post-GWAS analysis has allowed us to identify causal genetic variants in phenotypes at the SNP, locus, and gene levels [13], as well as genetic overlap with brain health-related traits [14]. The heritability analysis has also helped to clarify the genetically determined proportions of phenotypes and guided downstream analyses that model functional mechanisms and pathways.

**Fig. 1:**
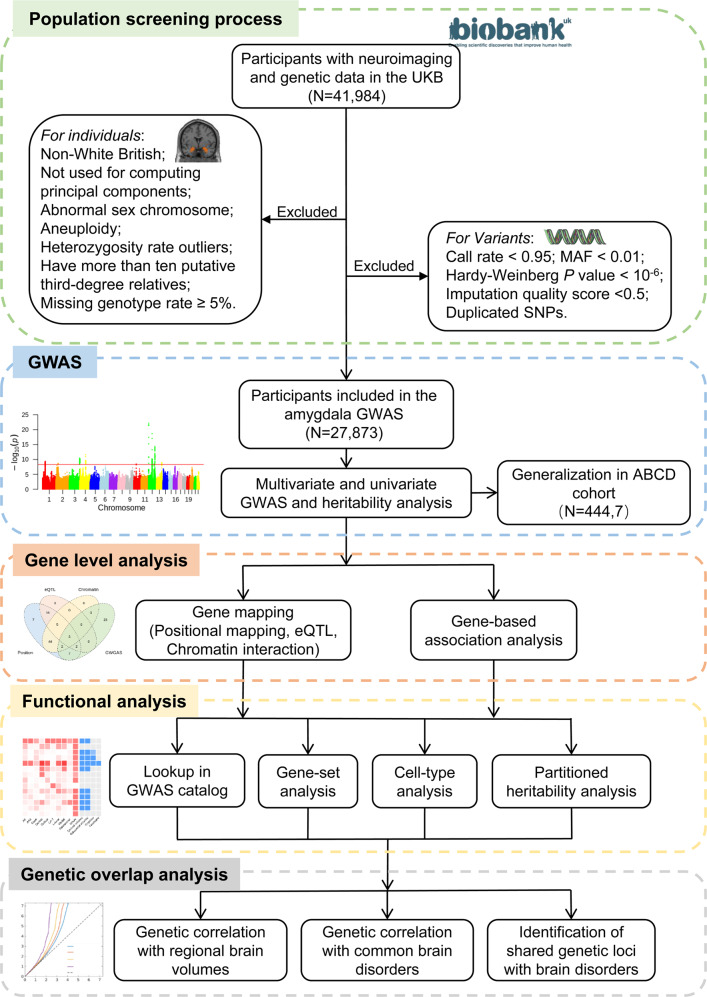
The schematic workflow of the study design. UKB UK biobank, MAF minor allele frequency, SNP single nucleotide polymorphism, GWAS genome-wide association study, ABCD Adolescent Brain Cognitive Development, eQTL expression quantitative trait loci.

In this study, we aimed to delve into the common variant influences of amygdala volume, their associations with altered gene expression, genetic overlap with other brain phenotypes, and, importantly, significant associations with neurological and psychiatric disorders. The whole amygdala was segmented into nine nuclei groups using Bayesian amygdala segmentation [2] (Methods). First, we started our analysis by performing a multivariate GWAS to discover the genetic variants of the amygdala using UKB participants [15]. For comparison, we also conducted the univariate GWAS of each of the amygdala nuclei. The SNP-based heritability, representing the phenotypic variance that arises due to the genetics, was also estimated [16]. Second, we discovered gene-level and gene-set associations, explored the functional mechanisms of the significant SNPs, and performed association lookups within the NHGRI-EBI GWAS catalog [17]. Third, we calculated the pairwise genetic correlations via linkage disequilibrium score (LDSC) regression with other regional brain volumes [18] and performed the cell-type specificity of the annotated genes. Finally, we detected the genetic overlap between ten volumes of the amygdala regions and ten psychiatric and neurological disorders. In addition, we analyzed the UKB GWAS results with those from the Adolescent Brain Cognitive Development (ABCD) study, which differed substantially from UKB in terms of age, therefore providing a powerful test of generalizability of the reported relevance. Collectively, our goal is to enable critical breakthroughs into the genetic architecture of human amygdala nuclei and substantiate the emerging view of the amygdala having crucial roles in common brain disorders. The schematic workflow is illustrated in Fig. 1.

## Materials and methods

### Amygdala segmentation and quality control procedures

We used the brain MRI data of 41,984 genotyped individuals from the UKB (http://www.ukbiobank.ac.uk/) [19] under accession number 19542. UKB has received ethical approval from the National Health Service National Research Ethics (ref: 11/NW/0382) and obtained informed consent from its participants. The MRI data were segmented into the whole amygdala and nine amygdala nuclei groups, accessory basal nucleus, anterior amygdaloid area-AAA, basal nucleus, central nucleus, cortical nucleus, corticoamygdaloid transition, lateral nucleus, medial nucleus, and paralaminar nucleus, using Bayesian amygdala segmentation [2]. In short, the segmentation of the amygdala is a Bayesian inference problem within a generative model of MRI images [2]. The segmentation algorithm minimizes the cost function by alternately optimizing the deformation of the atlas mesh and the Gaussian parameters (means and variances), which were estimated directly from the MRI scan [2]. The generalization sample comprised MRI and SNP data from the ABCD cohort collected from 21 acquisition sites. The T1-weighted scans were collected using Siemens Prisma, GE 750, and Philips 3T scanners [20]. All procedures were approved by the Institutional Review Boards. Parents or guardians provided written informed consent, and children assented before participation.

Our analytic sample was restricted to white British individuals whose data were used in calculating principal components (PCs). We applied standard quality control (QC) procedures to the UKB v3 imputed genetic data by removing SNPs with call rate < 0.95, imputation quality score < 0.5, a minor allele frequency (MAF) < 0.01, failing the Hardy–Weinberg equilibrium tests at *P* < 1 × 10^−06^, or duplicated, and further filtered out individuals with abnormal sex chromosome, putative sex chromosome aneuploidies, heterozygosity rate outliers, having more than ten putative third-degree relatives, or missing genotype rate ≥ 5% using PLINK [21]. For ABCD, we downloaded the genetic data from the third release and subsequently applied similar post-imputation QC procedures. Individuals with non-European ancestry, missing genotype rate exceeding 0.05, a heterozygosity rate deviating more than 3 SD from the mean, or relatedness (PiHat > 0.2) and variants with call rate < 0.95, MAF < 0.01, Hardy–Weinberg *P* value < 1 × 10^−06^ were removed before imputation. We imputed the ABCD genetics data to the HRC panel (v1.1 2016) through Michigan Imputation Server with Eagle v2.4 phasing. After QC, the final analytic sample size in UKB and ABCD were 27,866 and 4,447, respectively.

### Multivariate and univariate genetic association analyses and identification of genomic loci

Amygdala estimates of both hemispheres were summed together to reduce the number of analyses. GWAS was run for the whole amygdala volume and the nine nuclei via PLINK 1.9 [21]. Covariates included age, age^2^, sex, scanning site, intracranial volume (ICV), and the first ten genetic PCs. The resulting residuals for the 10 regions were jointly fed into the multivariate omnibus statistical test (MOSTest) analysis [11]. MOSTest implements permutation testing to identify genetic effects across multiple phenotypes [22], yielding a multivariate GWAS summary statistic across all 10 features. For comparison, we also performed univariate GWAS of the nine nuclei and the whole amygdala. Besides the basic covariates, the GWAS for the nine nuclei was run both with and without accounting for whole amygdala volume.

Genomic risk loci were identified by the Functional Mapping and Annotation (FUMA) of GWAS SNP2GENE online platform [23] (version 1.3.7, http://fuma.ctglab.nl/). Allele LD correlations were computed from the European panel of the 1000 Genomes phase 3 data. Independent significant SNPs were identified by the statistical threshold (5 × 10^−08^) and independency (r^2^ ≤ 0.6) [24]. Lead variants were defined as those significant variants independent of each other at r^2^ < 0.1 [24]. Candidate SNPs were defined as all SNPs in LD (r^2^ ≥ 0.6) with one of the independent significant SNPs in the genetic loci [24]. Genomic loci were characterized by merging LD blocks that are located close to each other (< 250 kb apart) [24].

The NHGRI-EBI GWAS catalog [17] was subsequently searched for independent significant SNPs and relevant SNPs (SNPs in LD with them) to look for reported associations with any other traits. We mainly focused on traits related to brain imaging, cognitive functions (e.g., general cognitive ability), neurodegenerative diseases (e.g., AD and PD), and neuropsychiatric disorders (e.g., major depression disorder [MDD], SCZ, and autism spectrum disorder [ASD]).

### Gene mapping, gene-based association, and gene-set analysis

FUMA [23] annotates significantly amygdala-linked SNPs with functional categories, including Combined annotation-dependent depletion (CADD) scores [25], RegulomeDB scores [26], and 15-core chromatin states [23], using a hypergeometric test. A CADD score above 12.37 is suggestive of a deleterious protein effect [25], whereas a lower RegulomeDB score indicates a higher probability of regulatory function. Categories 1–7 of chromatin states are considered open chromatin states [27]. Positional, expression quantitative trait loci (eQTL), and 3D chromatin interaction mappings [23] were used to map all of the independent significant variants to genes. We used default values for all of the parameters and applied FDR of 0.05 to define significant associations.

Genome-wide gene-based association analyses (GWGAS) analysis was performed using GWAS summary statistics as input into Multimarker Analysis of GenoMic Annotation (MAGMA) (v1.08) [28] with default settings, which made use of the European panel of the 1000 Genomes phase 3 data as the reference. The major histocompatibility complex (MHC) region was excluded before the analysis. The Bonferroni-corrected significant threshold was *P* = 0.05/18,879 genes = 2.65 × 10^−06^. In addition, we performed a gene-set analysis using the g:Cocoa (compact comparison of annotations) function in g:Profiler web server for curated gene sets, gene-ontology (GO) terms, biological pathways, and protein databases.

### Cell specificity analysis and genetic correlations with brain volumes

To assess whether genes are disproportionately expressed in certain cell types, we investigated associations with several gene expression profiles using MAGMA’s CELL TYPE function in FUMA. FDR corrected *P* values < 0.05 were considered significant. Additionally, we used LDSC [18] to estimate the pairwise genetic correlations (*rg*) between amygdala volume and 101 brain volumes reported by Zhao et al.’s GWAS [29]. Genetic correlations for which the *P* value survived the FDR correction (*P* < 0.05) were considered significant.

### SNP-based heritability and partitioned heritability estimation

SNP-based heritability analyses were conducted using LDSC regression [18]. Heritability describes the proportion of phenotypic variance explained by genetic variance, in which genomic inflation factors (λ_GC_), LDSC intercepts, and LDSC ratios for each GWAS were calculated. We used precomputed LD scores calculated by 1000 Genomes European data. Partitioned heritability refers to the proportion of heritability explained by annotated regions of the genome into 97 functional classes, which is estimated by the partitioned LDSC [30] method. We focused on enriched annotations where regression coefficients are significantly positive [31] (z > 1.96, two-tailed *P* < 0.05).

### Genetic overlap between amygdala volumes and ten brain disorders

To further examine the genetic overlap between amygdala regions and ten psychiatric and neurological disorders, GWAS summary statistics for AD [32], attention deficit hyperactivity disorder (ADHD) [33], anxiety disorders [34], ASD [35], bipolar disorder (BD) [36], Lewy body dementia (LBD) [37], MDD [38], motor subtypes of PD [39], PTSD [40], and SCZ [41] were obtained. LDSC regression analyses were performed. We also conducted the latent causal variable (LCV) analysis [42], under which the genetic correlation between two traits is mediated by a latent variable having a causal effect on each trait. We quantify partial causality using the genetic causality proportion. Informally, the LCV model assumes that any asymmetry in the shared genetic architecture arises from the action of a latent variable.

cFDR (condFDR and conjFDR) methods using MATLAB R2018b and Python 3.7.7 were employed. Using the associations between genetic variants and the secondary phenotype, the condFDR analysis re-ranked test statistics and recalculated the associations between these variants and the primary phenotype, thus prioritizing variants for follow-up analyses [43]. We plotted the empirical cumulative distribution of nominal *P* values for all SNPs in one phenotype (e.g., whole amygdala volume) and for subsets of SNPs with significance levels in another phenotype (e.g., SCZ) below the indicated cutoffs (*P* ≤ 1, *P* ≤ 0.1, *P* ≤ 0.01, and *P* ≤ 0.001). The enrichment is visualized as successive leftward deflections from the null distribution in conditional quantile-quantile (Q-Q) plots. We further made use of the conjFDR [44] method, which is an extension of condFDR and defined by the maximum of the two condFDR values for a specific SNP, to detect the genetic loci shared between traits. Regions of complex LD patterns, such as MHC (chr 6: 25119106-33854733) and 8p23.1 (chr 8: 7242715-12483982) regions, APOE for AD, and MAPT for PD were excluded before performing the analysis. The FDR significance cutoffs were 0.01 for condFDR and 0.05 for conjFDR, in line with prior studies [13, 45].

## Results

### Multivariate and univariate GWAS of amygdala volumes

**Fig. 2:**
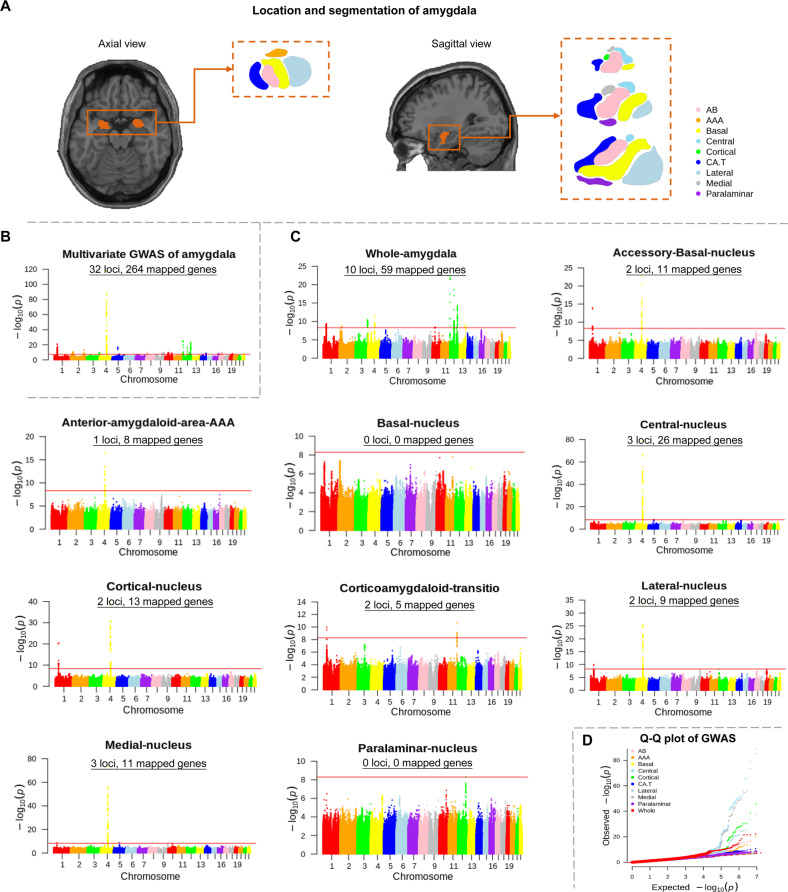
Multivariate and univariate GWAS of amygdala volumes. **A** The location of the amygdala region in the human brain (colored in orange) and the subdivisions of the amygdala from the sagittal and axial view. **B** Multivariate GWAS for amygdala volumes. This part illustrates the -log10(*P*) statistic from the multivariate GWAS across the entire formation, with 32 significant loci. The GWAS significant threshold is set at *P* < 5 × 10^−08^. **C** Manhattan plots of genetic variants underlying univariate GWAS of amygdala volumes. Number of significant genetic loci and mapped genes are listed in Manhattan subplot titles, with the horizontal red line denoting GWA significance (*P* < 5 × 10^−09^). **D** Q-Q plot of the GWAS for the amygdala volumes. The Q-Q plot showed that potential population stratification and/or cryptic relatedness are well controlled after genomic correction. GWAS genome-wide association study, Q-Q quantile-quantile, AB accessory-basal nucleus, AAA anterior-amygdaloid-area, CAT corticoamygdaloid transition.

The GWAS made use of data of 27,866 UKB brain imaged samples (52.1% females; age range: 46–82 years; Table 1), accounting for age, age^2^, sex, imaging site, ICV, and the first ten genetic PCs. The location of the amygdala region in the human brain and the nine amygdala nuclei were depicted in Fig. 2A.

**Table 1:** Samples and phenotype descriptions.

Characteristics	Discovery (UKB)	Generalization (ABCD)
Total *N*	27,866	4447
Age (mean ± SD)	64.28 ± 7.50	9.90 ± 0.70
Age (range)	46–82	9–11
Female, *N* (%)	52.10%	46.98%
Amygdala phenotypes		
Whole amygdala (mm^3^)	1702.55 ± 192.89	1620.08 ± 211.62
Accessory-Basal nucleus (mm^3^)	254.55 ± 31.88	NA
Anterior-amygdaloid-area-AAA (mm^3^)	50.47 ± 7.00	NA
Basal nucleus (mm^3^)	424.98 ± 50.43	NA
Central nucleus (mm^3^)	50.48 ± 8.88	NA
Cortical nucleus (mm^3^)	26.79 ± 4.67	NA
Corticoamygdaloid transition (mm^3^)	170.31 ± 23.24	NA
Lateral nucleus (mm^3^)	651.93 ± 74.56	NA
Medial nucleus (mm^3^)	21.94 ± 5.58	NA
Paralaminar nucleus (mm^3^)	51.10 ± 6.14	NA

*UKB* UK biobank, *ABCD* Adolescent Brain Cognitive Development, *SD* standard deviation, *NA* not available.

**Fig. 3:**
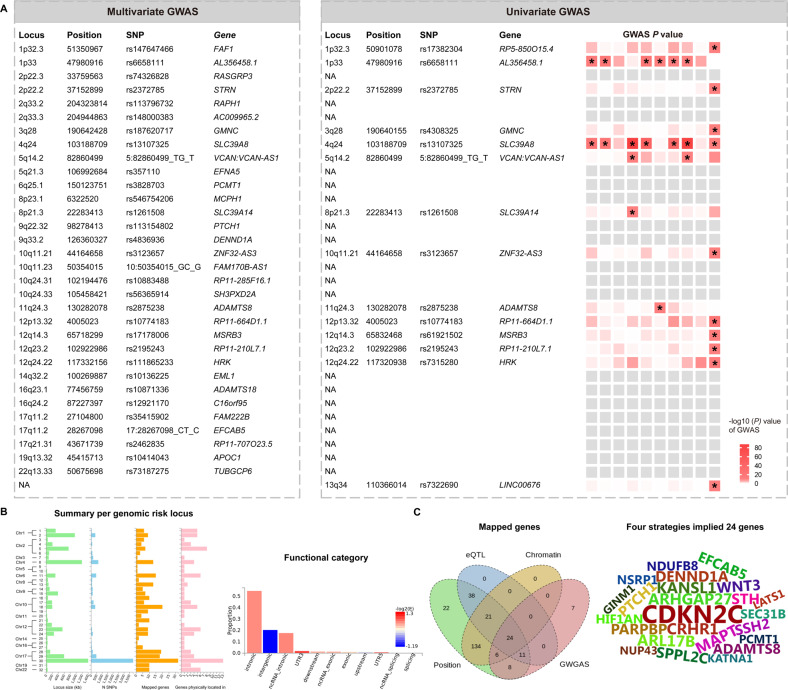
The comparison of genetic loci between the multivariate and univariate GWAS, and functional annotation. **A** The left column indicates the 32 genetic loci identified by the multivariate GWAS, whereas the right column indicates the 14 loci identified by the univariate GWAS. The heatmap shows, from left to right, accessory basal nucleus, anterior amygdaloid area-AAA, basal nucleus, central nucleus, cortical nucleus, corticoamygdaloid transition, lateral nucleus, medial nucleus, paralaminar nucleus and whole amygdala volumes. Significant loci in the univariate GWAS (*P* < 5 × 10^−09^) are marked with an asterisk *. 13 of the 14 loci identified in the univariate GWAS were replicated in the multivariate GWAS, except the 13q34 locus specific for the whole amygdala volume. **B** Functional annotation of the genomic loci and identified SNPs. Left panel, overview of the genomic loci sizes and number of variants. Right panel, distribution of functional consequences of SNPs in significant genomic loci. **C** Gene mapping consequences of the significant SNPs. Left panel, venn diagram showing the number of genes mapped by the four different strategies, i.e., positional (green), eQTL (blue), chromatin interaction mapping (yellow), and identification by the GWGAS (red). A total of 271 genes were identified by four approaches. Right panel, 24 genes were identified by all of the four mapping strategies, showing by the wordcloud plot. GWAS genome-wide association study, SNP single nucleotide polymorphism, eQTL expression quantitative trait loci.

The multivariate GWAS identified 32 genetic loci and 98 independent significant SNPs under *P* < 5 × 10^−08^ (Fig. 2B, Supplementary Table S1). The univariate GWAS additionally accounting for the whole amygdala volume, identified significant hits for 8 of the 10 volumes and a total of 76 independent significant SNPs, tagging 14 independent genomic loci after correcting for the number of traits analyzed (Bonferroni-corrected *P* < 5 × 10^−08^/10 = 5 × 10^−09^) (Fig. 2C, Supplementary Table S2). The Q-Q plots depicted that potential population stratification and/or cryptic relatedness are well controlled after genomic correction (Fig. 2D). Specifically, 10 of the 14 genetic loci were associated with the whole amygdala; 2, 1, 3, 2, 2, 2, and 3 loci were associated with the volumes of accessory basal, anterior-amygdaloid-area-AAA, central, cortical, corticoamygdaloid transition, lateral, and medial nucleus, respectively. 11 of the 14 genetic risk loci were associated with only one volume; within 9 of them were associated with the whole amygdala volume, 1 with the central nucleus, and 1 with the corticoamygdaloid transition. 4 of these unique genetic loci were associated with volumes of the individual nuclei and not with whole volume. Overall, 13 of the 14 loci identified in the univariate GWAS were replicated in the multivariate GWAS, except the rs7322690 in the 13q34 specific for whole amygdala volume (Fig. 3A).

The set of nuclei GWAS without co-varying for whole amygdala volume identified a total of 19 loci over nine nuclei (Supplementary Table S3). A total of 4, 4, 3, 5, 3, 4, 11, 4 and 4 loci were associated with the volumes of accessory basal, AAA, basal, central, cortical, corticoamygdaloid transition, lateral, medial, and paralaminar nucleus, respectively.

### Generalization in ABCD cohort

The amygdala-associated lead SNPs of the UKB sample were further evaluated in the generalization GWAS of the ABCD cohort (*N* = 4,447; 47.0% females; age range: 9–11 years), in which only the data on whole amygdala volume was available. We found that 13 out of the 21 independent significant SNPs from the discovery GWAS had the same effect direction in generalization (Supplementary Table S2). Moreover, 6 of the discovery lead SNPs had uncorrected *P* < 0.05, whereas 15 had uncorrected *P* > 0.05 in the generalization cohort. The locus of 12q23.2 was also discovered in ABCD (Supplementary Table S4). The nearest gene of this locus is *RP11-210L7.1*, one of the intergenic long non-coding RNAs (lncRNAs), however, have not been annotated.

### Functional annotation and gene-based association

A majority of these independent significant SNPs were significantly enriched for noncoding regions, i.e., 52.0% for intronic, and 30.6% for intergenic (Fig. 3B, Supplementary Table S1). About 88.8% of the SNPs had a minimum chromatin state of 1–7, indicating a location within regulatory regions. CADD scores indicated 5 lead SNPs (rs17248480, rs13107325, rs4836936, rs17178006, rs113562005) as pathogenic with scores > 12.37. Positional, eQTL, and chromatin interaction mapping were employed in FUMA [23] to map candidate SNPs to genes (Supplementary Tables S5-7). We next conducted GWGAS using MAGMA [28] and detected 59 unique genes (*P* < 2.65 × 10^−06^; Supplementary Table S8). These four strategies identified 271 unique genes, where 29 were implicated by one mapping strategy, 172 genes by two strategies, 46 by three strategies, and 24 by all of the four types of gene mapping (Fig. 3C). The list of the 24 genes was depicted in the wordcloud plot, including the carcinoma-related gene *cyclin dependent kinase inhibitor 2C* (*CDKN2C)* [46], tau-encoding gene *Microtubule associated protein tau* (*MAPT)* [47], and gene participating in neuronal plasticity, *ADAM metallopeptidase with thrombospondin type 1 motif 8 (ADAMTS8)* [48].

Gene-set analysis implicated by MAGMA revealed several significant gene sets involved in cell differentiation/development, and axon extension functions (Supplementary Table S9). Further functional enrichment analysis using g:Profiler web server identified significant enrichment within various ion transport-related sets for the majority of the amygdala regions (Supplementary Table S10).

### SNP-based heritability and partitioned heritability analysis

**Fig. 4:**
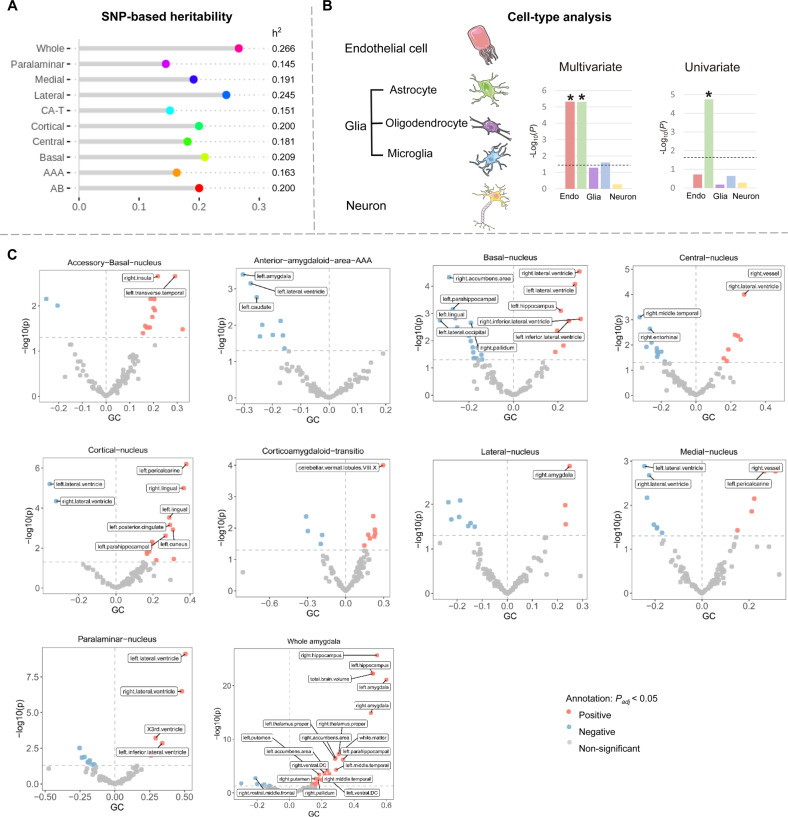
Heritability estimates and genetic overlap with brain structures. **A** LDSC-based heritability estimates for the amygdala volumes. All amygdala volumes showed substantial heritability, ranging from 0.145 for corticoamygdaloid transition to 0.266 for the whole amygdala. **B** Cell-type analysis indicated that most of the genes were significantly enriched in astrocytes. Left panel represents the significant association for cell-type analysis was observed for endothelial cells and astrocytes in the multivariate GWAS. Right panel represents that most of the genes were significantly enriched in astrocytes for the whole amygdala volume in the univariate GWAS. **C** Volcano plots visualize the genetic correlation estimates between 101 regional brain volumes and accessory-basal, anterior-amygdaloid-area-AAA, basal, central, cortical, corticoamygdaloid transition, lateral, medial, paralaminar nucleus, and whole amygdala volume. Red, blue, and gray dots indicate positive, negative, and non-significant genetic associations, respectively. Correlation estimates that survived FDR adjustment are annotated with brain region names. SNP single nucleotide polymorphism, AB accessory-basal nucleus, AAA anterior-amygdaloid-area, CAT corticoamygdaloid transition, LDSC linkage disequilibrium score, FDR false discovery rate.

SNP-based heritability (h^2^) was 27% for the whole amygdala and ranged from 14% to 25% for the nine nuclei, illustrating themselves as genetically determined regions (Fig. 4A, Supplementary Table S11). The LDSC intercepts were close to 1, suggesting that the observed inflation in genetic signal is mostly due to polygenic signal instead of population stratification [49].

We focused on enriched annotations where regression coefficients are significantly positive (z > 1.96, two-tailed *P* < 0.05; Supplementary Table S12) in stratified LDSC regression. Specifically, we found notable enrichment for three phylogenetically conserved cortical regions (i.e., enrichment = 22.63, *P* = 0.029 for accessory basal; 26.72, 0.018 for corticoamygdaloid transition and 24.96, 0.033 for paralaminar nucleus) for the annotation “ancient sequence age human promoter”. This conserved promoter annotation represents a genomic region that predates the evolutionary split of marsupial and placental mammals [50].

### Concordance with previous studies

Most of these loci were previously reported to be related with brain volume and white matter microstructure traits (Supplementary Table S13). We noted that 4q24 was related with cognitive performances, SCZ, and BD, 9q22.32 with cognitive traits and depression, 17q21.31 with cognitive traits, PD and SCZ, and 19q13.32 with cognitive performances, AD, and LBD. However, 11 of the 32 loci have not been reported to be related to imaging or cognitive/psychiatric traits before, namely the loci of 2q33.3, 2q33.2, 5q21.3, 8p23.1, 8p21.3, 10q11.21, 10q24.31, 14q32.2, 16q23.1, 17q11.2, and 22q13.33.

We subsequently compared our univariate GWAS results with those reported by Zhao et al. [29] and Smith et al. [12] (Supplementary Table S14). 21 and 18 of the 21 independent significant variants were supported to be significantly associated with left and right amygdala volumes in the Zhao et al.’ s study, respectively. 7 were replicated in the GWAS by Smith et al. (*P* < 0.05).

### Cell-type analysis and genetic overlap with brain volumes

We found that the genes of multivariate GWAS was enriched in astrocytes (*P*_FDR_ = 2.23 × 10^−04^ in PsychENCODE_Adult database), and endothelial cells (*P*_FDR_ = 2.23 × 10^−04^ in PsychENCODE_Adult database; Fig. 4B and Supplementary Table S15). For the univariate GWAS, the significant evidence of association for whole amygdala volume cell-type analysis was observed for astrocytes (*P*_FDR_ = 0.026 in PsychENCODE_Adult database). Astrocytes are the most abundant cell type in the brain, playing vital roles in governing key steps in synapse formation and plasticity [51].

Next, we used the GWAS results to estimate the genetic overlap with brain volumetric traits via LDSC [18] (Supplementary Table S16 and Fig. 4C). Significant genetic correlations (*P*_FDR_ < 0.05) were observed between whole amygdala volumes and 21 traits, amongst which were positive genetic correlations with hippocampus (right: *rg* = 0.60, *P*_FDR_ = 2.6 × 10^−29^; left: *rg* = 0.56, *P*_FDR_ = 9.5 × 10^−23^), amygdala (right: *rg* = 0.62, *P*_FDR_ = 2.7 × 10^−20^; left: *rg* = 0.68, *P*_FDR_ = 2.8 × 10^−26^), thalamus (right: *rg* = 0.37, *P*_FDR_ = 1.3 × 10^−07^; left: *rg* = 0.34, *P*_FDR_ = 3.2 × 10^−07^), accumbens area (right: *rg* = 0.32, *P*_FDR_ = 3.2 × 10^−06^; left: *rg* = 0.22, *P*_FDR_ = 7.5 × 10^−03^), putamen (right: *rg* = 0.23, *P*_FDR_ = 3.9 × 10^−03^; left: *rg* = 0.25, *P*_FDR_ = 1.2 × 10^−03^) and middle temporal (right: *rg* = 0.29, *P*_FDR_ = 3.6 × 10^−03^; left: *rg* = 0.33, *P*_FDR_ = 1.3 × 10^−03^), and reverse correlations with right lateral ventricle (*rg* = −0.20, *P*_FDR_ = 0.030).

### Genetic overlap with ten common brain disorders

**Fig. 5:**
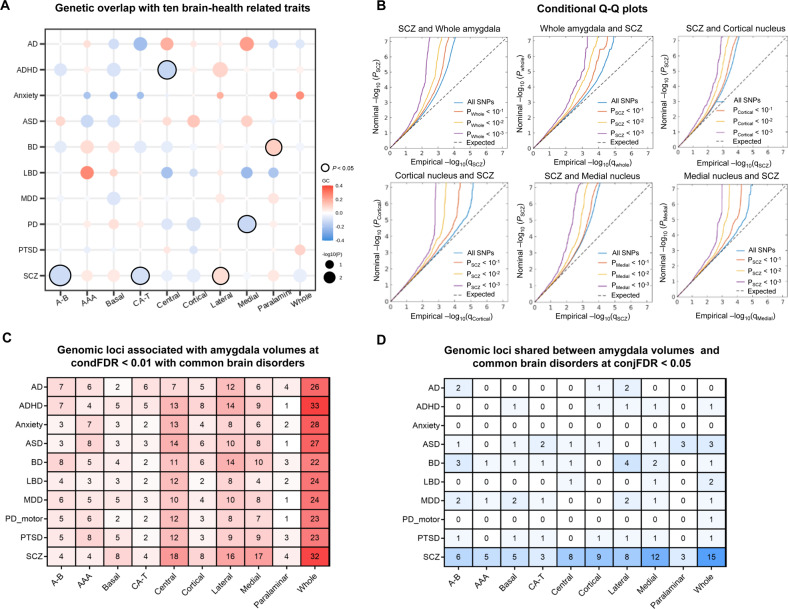
Genetic overlap between amygdala volumes and ten common brain disorders. **A** Genetic correlations between amygdala volumes and ten brain-health-related traits were assessed using LDSC regression. Warm and cool colors indicate positive and negative associations, respectively. There were significant positive correlations between the lateral nucleus and SCZ, paralaminar nucleus and BD; negative correlations between the accessory-basal nucleus, corticoamygdaloid transition and SCZ, central nucleus and ADHD, medial nucleus and PD, as indicated by a black frame. No associations passed the FDR correcting across all 100 analyses (10 volumes × 10 traits; two-sided *P*_FDR_ < 0.05). **B** Conditional Q-Q plots for whole amygdala volume, cortical nucleus, and medial nucleus conditioned on SCZ and vice versa, demonstrating genetic overlap. **C** Enhanced discovery of genetic loci for each of the amygdala volumes when condFDR analyses were run for each of the amygdala volumes conditioned on the ten brain disorders. **D** ConjFDR analysis detected shared genetic loci across amygdala volumes and the ten clinical conditions. LDSC linkage disequilibrium score, AB accessory-basal nucleus, AAA anterior-amygdaloid-area, CAT corticoamygdaloid transition, AD Alzheimer’s disease, ADHD attention-deficit/hyperactivity disorder, ASD autism spectrum disorders, BD bipolar disorders, LBD Lewy body dementia, MDD major depression disorders, PD Parkinson’s disease, PTSD Post-traumatic stress disorder, SCZ schizophrenia, Q-Q quantile-quantile, FDR false discovery rate, condFDR conditional FDR, conjFDR conjunctional FDR.

Three nuclei showed significant consistent genetic correlations across the genome with SCZ (*rg* = −0.12, *P* = 0.002, for accessory basal nucleus; −0.10, 0.017 for corticoamygdaloid transition; 0.08, 0.031 for lateral nucleus). Moreover, apparent relationships were seen for central nucleus and ADHD (*rg* = −0.14; *P* = 0.018), medial nucleus and PD (*rg* = −0.13, *P* = 0.025), paralaminar nucleus and BD (*rg* = 0.11, *P* = 0.037). However, none of the correlations passed the FDR correction (Supplementary Table S17 and Fig. 5A). The LCV analysis showed that none of the ten association pairs reached statistical significance (Supplementary Table S18).

We leveraged the genetic overlap to discover more genetic underpinnings of amygdala volumes by employing condFDR statistics (Supplementary Table S19). The conditional Q-Q plots indicated successive increments of SNP enrichment for whole amygdala, cortical and medial nucleus volumes (Fig. 5B), consistent with polygenic overlap across volumes and disorders. We discovered a total of 33 genetic loci for the whole amygdala, and 8, 8, 8, 18, 8, 6, 16, 17, and 4 loci for the accessory basal, AAA, basal, central, cortical, corticoamygdaloid transition, lateral, medial, paralaminar nucleus, and whole amygdala, separately (Fig. 5C). We further performed conjFDR analysis, which enables the detection of genetic loci shared between traits (Supplementary Table S20). ConjFDR analysis with a threshold of 0.05 revealed 12 of the 15 GWAS signals showing evidence for a pleiotropic relationship with SCZ, with 3 GWAS signals jointly associated with more than one psychiatric condition: 1 with ADHD, ASD, BD, LBD, MDD, motor symptoms of PD, PTSD, and SCZ, 1 with ASD, LBD and SCZ, and 1 with ASD and SCZ (Fig. 5D).

## Discussion

Using brain scan data from 27,866 UKB white British participants, we analyzed the genetic architectures of amygdala volume and its nine nuclei, along with their roles in common brain disorders. Overall, via multivariate GWAS, we identified 32 independent genomic loci across 16 chromosomes and found moderate heritability for amygdala volumes. *RP11-210L7.1* in 12q23.2 locus was found to be closely related to amygdala. Bioinformatics analyses revealed enrichment of many biological pathways (mainly, cell differentiation/development and ion transporters), as well as cell-specific (astrocytes) functional enrichments. Evidence for pleiotropy of identified variants with SCZ was identified. Together, these results shed light on the genetic architecture, the biological functions, and the roles of the amygdala in the development of common brain disorders.

Amygdala is composed of multiple interconnected nuclei that exhibit distinctive functions, but there have yet to be large scale neuroimaging studies investigating the genetic underpinnings of these structures. We identified statistically significant associations between amygdala volume and variants at 32 genomic loci by the multivariate GWAS. Notably, 13 of the 14 genomic loci in the univariate GWAS were replicated in the multivariate analysis. For the univariate GWAS, the majority (10 of 14) of amygdala-linked loci were associated with only one of the ten volumes. Four of them were related to volumes of the individual amygdala regions but not the whole amygdala volume, illustrating the importance of targeting individual nuclei. For example, a significant association was observed at 5q14.2 within non-coding RNA *VCAN-AS1* for the central nucleus. This gene was reported to participate in the regulation of developing gastric [52] and breast cancer [53]. The lead SNP rs2875238 at 11q24.3 was identified to be associated with corticoamygdaloid transition solely, mapping to the *ADAMTS8* gene, which was found to have modifying effects on perineuronal net structures and involved in the formation of neuronal plasticity and long-term memory [48]. The discovery of novel loci might also help disentangle the potential associations between other specific diseases and brain disorders, in which we described 11 loci that have not been previously reported to be related to imaging or cognitive/psychiatric traits. For instance, the 8p21.3 locus with lead SNP rs1261508 within the *SLC39A14* gene, which is mainly expressed in cell membrane but also intracellularly in hepatocytes and large neurons [54]. Dysfunction of SLC39A14 would impair hepatic manganese uptake [54], and promote rapidly progressive childhood-onset parkinsonism-dystonia [55]. Notably, the locus of 12q23.2 was discovered in the univariate, multivariate GWAS of UKB, and generalized in the ABCD cohort. The nearest gene of this locus is *RP11-210L7.1*, one of the intergenic lncRNAs, however, have not been annotated. As we known, lncRNAs are transcribed and processed like mRNAs but do not code for functional proteins [56], and they play important roles in brain development, neuron function and maintenance, and neurodegenerative diseases [57]. In addition, neuroimaging studies have documented that the volumes of the amygdala are essentially heritable across the lifespan [9, 10]. We demonstrated similar SNP-based heritability estimates reported by Smith et al. [12], with ten volumes ranging from 0.15 to 0.27, despite it being lower than the estimates indicated in twin studies [10, 58]. Nevertheless, the differences may be derived from the upward bias due to gene-gene/gene-environment interactions in twins studies [59] or downward bias caused by the genetic influences not being fully captured by additive effects of common genetic variants [60].

The amygdala morphology and associated disorders have been further examined with the pathways modeling. We conducted functional annotation, gene-based, and gene-set analyses of each GWAS signal to identify likely causal SNPs. Notably, 24 genes were identified by all four gene mapping types, including *ADAMTS8* mentioned above. The indicated *CDKN2C* was reported to be correlated to carcinomas, especially thyroid-related disorders [46]. *MAPT* is a gene responsible for encoding tau protein, and has been presumed to play a crucial role in pathogenesis of neurodegenerative disorders, including frontotemporal dementia, AD, progressive supranuclear palsy, and PD [47]. Several SNPs had CADD scores >15, suggesting deleterious protein effects. For example, rs13107325 (score = 23.1) was associated with seven of the ten volumes located within the metal ions transporter gene *SLC39A8* on chromosome 4, which has previously been linked to PD [61] and SCZ [61]. rs17178006 on chromosome 12 was significant for whole amygdala volume (score = 18.91), and the gene nearest of which is *MSRB3*, which is associated with decreased hippocampal volume and increased risk of AD [62]. The gene-enrichment analysis highlighted “sodium: proton antiporter activity” (GO:0015385) pathway. Ion transporters play crucial roles in regulating ionic homeostasis, cell volume, and cellular signal transduction under physiological conditions in brain [63]. Genes that are part of this pathway have previously been implicated in various neurological diseases such as AD [64], PD [65], and multiple sclerosis [64].

Intriguingly, the regions of conserved genomic partitions were particularly enriched by the most statistically significant partitioned heritability, which was noted by the ancient sequence age human promotor, a conserved region that is evident to exist before the evolutionary split of mammals [50]. Using the LDSC regression method, we found positive genetic correlations between amygdala volumes and distinct subcortical volumetric measures, including hippocampus, thalamus, accumbens, and putamen; negative genetic correlations with the right lateral ventricle. These results suggest a strong genetic basis and inner link across subcortical structures. Even more supportive are the findings that the sets of identified genes showed the highest expression in astrocytes. Astrocytes are the most abundant cell type in the mammalian brain [66], playing an essential role in maintaining synapse function [51], blood-brain-barrier and ion homeostasis, and neurotransmission [67]. Astrocyte dysfunction has proven to be a common crossroads in neurodegenerative disorders (such as AD [68] and PD [69]) and psychiatric diseases (such as SCZ [70]). Subsequent studies with neuroimaging data across the life span are needed to validate these findings and determine genetic patterns in the nuclei of the amygdala.

The amygdala complex is composed of structurally and functionally distinct subregions that have critical yet differential roles in a range of disease states. We first identified the genetic overlap between amygdala volumes and ten neurological and psychiatric disorders via LDSC regression [18]. Though none of the association pairs passed the FDR correction, we still uncovered significant overlap with SCZ in three nuclei (i.e., accessory basal, corticoamygdaloid transition, and lateral nucleus). However, none of the ten association pairs reached statistical significance in the LCV causality analyses. We then searched for the shared genetic loci and the polygenic overlap for the amygdala regions in SCZ. The conjFDR analyses pinpointed a number of specific loci that overlapped, including *SLC39A8*, a gene well-known for its high pleiotropy, being associated with a range of traits besides SCZ, such as PD [61]. This locus was shared with AAA, accessory-basal, central, cortical, lateral, medial, and whole amygdala volume in SCZ. rs7792280 was shared with AB and lateral nucleus. The nearest gene for rs7792280 is a transcription factor-*SP4*, which is highly expressed in the brain and was reported to be related with an increased burden of rare deleterious mutations in SCZ [71]. rs7831557, shared between the central and lateral nucleus, encoding in *MSRA*, a member of the methionine-sulfoxide reductase system [72], was reported to be associated with an increased risk of BD and executive functions in a Han Chinese population [73]. Further mechanistic studies to elucidate how the amygdala contributes to the pathogenesis of brain disorders are warranted. These shared loci are noteworthy and could inform such explorations.

The present GWAS confronts some limitations. First, given that the UKB data were sampled from a cohort of British ancestry with a specific age range, the generalization of these findings to non-European ethnicities or specific clinical cohorts should be treated with caution. We hope diverse samples become increasingly available to further confirm our findings and provide novel discoveries. Second, the estimates of SNP-based heritability are narrow-sense, which only consider the additive genetic effects of common variants however ignore the effects of non-additive and rare loci as well as gene-gene/gene-environment interactions. Third, due to the lack of data on the amygdala segmentation in the ABCD cohort, we were unable to perform GWAS for each of the nuclei and the corresponding multivariate GWAS, and also the polygenic scores of each nucleus. Finally, since the bioinformatics tools relied on gene expression patterns in non-human samples or relatively small samples of postmortem human brains, the annotation results can only be considered suggestive rather than conclusive.

In summary, the current study provides new insights into the genetic architecture of amygdala regions by identifying the significant genetic loci through multivariate GWAS, functional annotating for biological processes, analyzing genetic overlap with other traits, and showing evidence for an involvement of amygdala regions in common brain disorders. Taken together, these results advanced our understanding of the genetic architecture of the amygdala and shed light on further research into the neurobiological basis of its anatomy and associations with brain disorders.

## Supplementary information

Supplementary Table

## Data Availability

Our GWAS summary statistics for the ten amygdala volumes can be accessed at https://figshare.com/articles/dataset/Multivariate_and_univariate_GWAS_summay_statistics_for_amygdala_volumes/22014926. The individual-level imaging and genetic data used in the present study are available through UKB (https://www.ukbiobank.ac.uk/) and ABCD (https://abcdstudy.org/).

## Code availability

This study used openly available software and codes, specifically R (https://www.r-project.org/), PLINK (https://www.cog-genomics.org/plink/), IMPUTE (https://mathgen.stats.ox.ac.uk/impute/impute_v2.html), Michigan Imputation Server (https://imputationserver.sph.umich.edu/), MOSTEST (https://github.com/precimed/mostest), FUMA (https://fuma.ctglab.n/l), MAGMA (https://ctg.cncr.nl/software/magma/, also implemented in FUMA), GWAS Catalog (https://www.ebi.ac.uk/gwas/), g:Profiler (https://biit.cs.ut.ee/gprofiler/gost/), LDSC (https://github.com/bulik/ldsc/), LCV (https://github.com/lukejoconnor/LCV), and condFDR/conjFDR (https://github.com/precimed/pleiofdr/).

## References

[1] LeDoux J (2007). The amygdala. Curr Biol.

[2] Saygin ZM, Kliemann D, Iglesias JE, van der Kouwe AJW, Boyd E, Reuter M (2017). High-resolution magnetic resonance imaging reveals nuclei of the human amygdala: manual segmentation to automatic atlas. NeuroImage.

[3] Sah P, Faber ES, Lopez De Armentia M, Power J (2003). The amygdaloid complex: anatomy and physiology. Physiol Rev.

[4] Pi G, Gao D, Wu D, Wang Y, Lei H, Zeng W (2020). Posterior basolateral amygdala to ventral hippocampal CA1 drives approach behaviour to exert an anxiolytic effect. Nat Commun.

[5] Kim S, Kwon SH, Kam TI, Panicker N, Karuppagounder SS, Lee S (2019). Transneuronal propagation of pathologic α-synuclein from the gut to the brain models Parkinson’s Disease. Neuron.

[6] Kantarci K, Avula R, Senjem ML, Samikoglu AR, Zhang B, Weigand SD (2010). Dementia with Lewy bodies and Alzheimer disease: neurodegenerative patterns characterized by DTI. Neurology.

[7] Hu X, Song X, Yuan Y, Li E, Liu J, Liu W (2015). Abnormal functional connectivity of the amygdala is associated with depression in Parkinson’s disease. Mov Disord.

[8] Alexandra Kredlow M, Fenster RJ, Laurent ES, Ressler KJ, Phelps EA (2022). Prefrontal cortex, amygdala, and threat processing: implications for PTSD. Neuropsychopharmacology.

[9] Satizabal CL, Adams HHH, Hibar DP, White CC, Knol MJ, Stein JL (2019). Genetic architecture of subcortical brain structures in 38,851 individuals. Nat Genet.

[10] den Braber A, Bohlken MM, Brouwer RM, van ‘t Ent D, Kanai R, Kahn RS (2013). Heritability of subcortical brain measures: a perspective for future genome-wide association studies. NeuroImage.

[11] van der Meer D, Frei O, Kaufmann T, Shadrin AA, Devor A, Smeland OB (2020). Understanding the genetic determinants of the brain with MOSTest. Nat Commun.

[12] Smith SM, Douaud G, Chen W, Hanayik T, Alfaro-Almagro F, Sharp K (2021). An expanded set of genome-wide association studies of brain imaging phenotypes in UK Biobank. Nat Neurosci.

[13] Visscher PM, Wray NR, Zhang Q, Sklar P, McCarthy MI, Brown MA (2017). 10 years of GWAS discovery: biology, function, and translation. Am J Hum Genet.

[14] Bulik-Sullivan B, Finucane HK, Anttila V, Gusev A, Day FR, Loh PR (2015). An atlas of genetic correlations across human diseases and traits. Nat Genet.

[15] Sudlow C, Gallacher J, Allen N, Beral V, Burton P, Danesh J (2015). UK biobank: an open access resource for identifying the causes of a wide range of complex diseases of middle and old age. PLoS Med.

[16] Tang M, Wang T, Zhang X (2022). A review of SNP heritability estimation methods. Brief Bioinforma.

[17] Buniello A, MacArthur JAL, Cerezo M, Harris LW, Hayhurst J, Malangone C (2019). The NHGRI-EBI GWAS Catalog of published genome-wide association studies, targeted arrays and summary statistics 2019. Nucleic Acids Res.

[18] Bulik-Sullivan BK, Loh PR, Finucane HK, Ripke S, Yang J, Patterson N (2015). LD Score regression distinguishes confounding from polygenicity in genome-wide association studies. Nat Genet.

[19] Alfaro-Almagro F, Jenkinson M, Bangerter NK, Andersson JLR, Griffanti L, Douaud G (2018). Image processing and Quality Control for the first 10,000 brain imaging datasets from UK Biobank. NeuroImage.

[20] Casey BJ, Cannonier T, Conley MI, Cohen AO, Barch DM, Heitzeg MM (2018). The Adolescent Brain Cognitive Development (ABCD) study: Imaging acquisition across 21 sites. Dev Cogn Neurosci.

[21] Purcell S, Neale B, Todd-Brown K, Thomas L, Ferreira MA, Bender D (2007). PLINK: a tool set for whole-genome association and population-based linkage analyses. Am J Hum Genet.

[22] Bahrami S, Nordengen K, Shadrin AA, Frei O, van der Meer D, Dale AM (2022). Distributed genetic architecture across the hippocampal formation implies common neuropathology across brain disorders. Nat Commun.

[23] Watanabe K, Taskesen E, van Bochoven A, Posthuma D (2017). Functional mapping and annotation of genetic associations with FUMA. Nat Commun.

[24] Elvsåshagen T, Bahrami S, van der Meer D, Agartz I, Alnæs D, Barch DM (2020). The genetic architecture of human brainstem structures and their involvement in common brain disorders. Nat Commun.

[25] Kircher M, Witten DM, Jain P, O’Roak BJ, Cooper GM, Shendure J (2014). A general framework for estimating the relative pathogenicity of human genetic variants. Nat Genet.

[26] Boyle AP, Hong EL, Hariharan M, Cheng Y, Schaub MA, Kasowski M (2012). Annotation of functional variation in personal genomes using RegulomeDB. Genome Res.

[27] Ernst J, Kellis M (2012). ChromHMM: automating chromatin-state discovery and characterization. Nat Methods.

[28] de Leeuw CA, Mooij JM, Heskes T, Posthuma D (2015). MAGMA: generalized gene-set analysis of GWAS data. PLoS Comput Biol.

[29] Zhao B, Luo T, Li T, Li Y, Zhang J, Shan Y (2019). Genome-wide association analysis of 19,629 individuals identifies variants influencing regional brain volumes and refines their genetic co-architecture with cognitive and mental health traits. Nat Genet.

[30] Finucane HK, Bulik-Sullivan B, Gusev A, Trynka G, Reshef Y, Loh PR (2015). Partitioning heritability by functional annotation using genome-wide association summary statistics. Nat Genet.

[31] Makowski C, van der Meer D, Dong W, Wang H, Wu Y, Zou J (2022). Discovery of genomic loci of the human cerebral cortex using genetically informed brain atlases. Science.

[32] Kunkle BW, Grenier-Boley B, Sims R, Bis JC, Damotte V, Naj AC (2019). Genetic meta-analysis of diagnosed Alzheimer’s disease identifies new risk loci and implicates Aβ, tau, immunity and lipid processing. Nat Genet.

[33] Demontis D, Walters RK, Martin J, Mattheisen M, Als TD, Agerbo E (2019). Discovery of the first genome-wide significant risk loci for attention deficit/hyperactivity disorder. Nat Genet.

[34] Meier SM, Trontti K, Purves KL, Als TD, Grove J, Laine M (2019). Genetic variants associated with anxiety and stress-related disorders: a genome-wide association study and mouse-model study. JAMA Psychiatry.

[35] Grove J, Ripke S, Als TD, Mattheisen M, Walters RK, Won H (2019). Identification of common genetic risk variants for autism spectrum disorder. Nat Genet.

[36] Stahl EA, Breen G, Forstner AJ, McQuillin A, Ripke S, Trubetskoy V (2019). Genome-wide association study identifies 30 loci associated with bipolar disorder. Nat Genet.

[37] Chia R, Sabir MS, Bandres-Ciga S, Saez-Atienzar S, Reynolds RH, Gustavsson E (2021). Genome sequencing analysis identifies new loci associated with Lewy body dementia and provides insights into its genetic architecture. Nat Genet.

[38] Wray NR, Ripke S, Mattheisen M, Trzaskowski M, Byrne EM, Abdellaoui A (2018). Genome-wide association analyses identify 44 risk variants and refine the genetic architecture of major depression. Nat Genet.

[39] Alfradique-Dunham I, Al-Ouran R, von Coelln R, Blauwendraat C, Hill E, Luo L (2021). Genome-wide association study meta-analysis for Parkinson disease motor subtypes. Neurol Genet.

[40] Nievergelt CM, Maihofer AX, Klengel T, Atkinson EG, Chen CY, Choi KW (2019). International meta-analysis of PTSD genome-wide association studies identifies sex- and ancestry-specific genetic risk loci. Nat Commun.

[41] Pardiñas AF, Holmans P, Pocklington AJ, Escott-Price V, Ripke S, Carrera N (2018). Common schizophrenia alleles are enriched in mutation-intolerant genes and in regions under strong background selection. Nat Genet.

[42] O’Connor LJ, Price AL (2018). Distinguishing genetic correlation from causation across 52 diseases and complex traits. Nat Genet.

[43] Andreassen OA, Djurovic S, Thompson WK, Schork AJ, Kendler KS, O’Donovan MC (2013). Improved detection of common variants associated with schizophrenia by leveraging pleiotropy with cardiovascular-disease risk factors. Am J Hum Genet.

[44] Liu JZ, Hov JR, Folseraas T, Ellinghaus E, Rushbrook SM, Doncheva NT (2013). Dense genotyping of immune-related disease regions identifies nine new risk loci for primary sclerosing cholangitis. Nat Genet.

[45] Elvsåshagen T, Shadrin A, Frei O, van der Meer D, Bahrami S, Kumar VJ (2021). The genetic architecture of the human thalamus and its overlap with ten common brain disorders. Nat Commun.

[46] Grubbs EG, Williams MD, Scheet P, Vattathil S, Perrier ND, Lee JE (2016). Role of CDKN2C copy number in sporadic medullary thyroid carcinoma. Thyroid Off J Am Thyroid Assoc.

[47] Zhang CC, Xing A, Tan MS, Tan L, Yu JT (2016). The role of MAPT in neurodegenerative diseases: genetics, mechanisms and therapy. Mol Neurobiol.

[48] Rossier J, Bernard A, Cabungcal JH, Perrenoud Q, Savoye A, Gallopin T (2015). Cortical fast-spiking parvalbumin interneurons enwrapped in the perineuronal net express the metallopeptidases Adamts8, Adamts15 and Neprilysin. Mol Psychiatry.

[49] Yang J, Weedon MN, Purcell S, Lettre G, Estrada K, Willer CJ (2011). Genomic inflation factors under polygenic inheritance. Eur J Hum Genet.

[50] Hujoel MLA, Gazal S, Hormozdiari F, van de Geijn B, Price AL (2019). Disease heritability enrichment of regulatory elements is concentrated in elements with ancient sequence age and conserved function across species. Am J Hum Genet.

[51] Freeman MR (2010). Specification and morphogenesis of astrocytes. Science.

[52] Feng L, Li J, Li F, Li H, Bei S, Zhang X (2019). Long noncoding RNA VCAN‐AS1 contributes to the progression of gastric cancer via regulating p53 expression. J Cell Physiol.

[53] Du P, Luo K, Li G, Zhu J, Xiao Q, Li Y (2021). Long non-coding RNA VCAN-AS1 promotes the malignant behaviors of breast cancer by regulating the miR-106a-5p-mediated STAT3/HIF-1α pathway. Bioengineered.

[54] Balint B, Bhatia KP (2016). SLC39A14 mutations expand the spectrum of manganese transporter defects causing parkinsonism-dystonia. Mov Disord.

[55] Tuschl K, Meyer E, Valdivia LE, Zhao N, Dadswell C, Abdul-Sada A (2016). Mutations in SLC39A14 disrupt manganese homeostasis and cause childhood-onset parkinsonism–dystonia. Nat Commun.

[56] Kleaveland B, Shi CY, Stefano J, Bartel DP (2018). A network of noncoding regulatory RNAs acts in the mammalian brain. Cell.

[57] Wu P, Zuo X, Deng H, Liu X, Liu L, Ji A (2013). Roles of long noncoding RNAs in brain development, functional diversification and neurodegenerative diseases. Brain Res Bull.

[58] Roalf DR, Vandekar SN, Almasy L, Ruparel K, Satterthwaite TD, Elliott MA (2015). Heritability of subcortical and limbic brain volume and shape in multiplex-multigenerational families with schizophrenia. Biol Psychiatry.

[59] Zuk O, Hechter E, Sunyaev SR, Lander ES (2012). The mystery of missing heritability: genetic interactions create phantom heritability. Proc Natl Acad Sci USA.

[60] Yang J, Zeng J, Goddard ME, Wray NR, Visscher PM (2017). Concepts, estimation and interpretation of SNP-based heritability. Nat Genet.

[61] Pickrell JK, Berisa T, Liu JZ, Ségurel L, Tung JY, Hinds DA (2016). Detection and interpretation of shared genetic influences on 42 human traits. Nat Genet.

[62] Adams SL, Benayoun L, Tilton K, Chavez OR, Himali JJ, Blusztajn JK (2017). Methionine sulfoxide reductase-B3 (MsrB3) protein associates with synaptic vesicles and its expression changes in the Hippocampi of Alzheimer’s disease patients. J Alzheimer’s Dis.

[63] Song S, Luo L, Sun B, Sun D (2019). Roles of glial ion transporters in brain diseases. Glia.

[64] Boscia F, Begum G, Pignataro G, Sirabella R, Cuomo O, Casamassa A (2016). Glial Na(+) -dependent ion transporters in pathophysiological conditions. Glia.

[65] Boscia F, D’Avanzo C, Pannaccione A, Secondo A, Casamassa A, Formisano L (2012). Silencing or knocking out the Na(+)/Ca(2+) exchanger-3 (NCX3) impairs oligodendrocyte differentiation. Cell Death Differ.

[66] Blanco-Suárez E, Caldwell ALM, Allen NJ (2017). Role of astrocyte-synapse interactions in CNS disorders. J Physiol.

[67] Guillamón-Vivancos T, Gómez-Pinedo U, Matías-Guiu J (2015). Astrocitos en las enfermedades neurodegenerativas (I): función y caracterización molecular. Neurolía.

[68] Carter SF, Herholz K, Rosa-Neto P, Pellerin L, Nordberg A, Zimmer ER (2019). Astrocyte Biomarkers in Alzheimer’s Disease. Trends Mol Med.

[69] Wei ZD, Shetty AK (2021). Treating Parkinson’s disease by astrocyte reprogramming: progress and challenges. Sci Adv.

[70] Dietz AG, Goldman SA, Nedergaard M (2020). Glial cells in schizophrenia: a unified hypothesis. lancet Psychiatry.

[71] Trubetskoy V, Pardiñas AF, Qi T, Panagiotaropoulou G, Awasthi S, Bigdeli TB (2022). Mapping genomic loci implicates genes and synaptic biology in schizophrenia. Nature.

[72] Moskovitz J, Jenkins NA, Gilbert DJ, Copeland NG, Jursky F, Weissbach H (1996). Chromosomal localization of the mammalian peptide-methionine sulfoxide reductase gene and its differential expression in various tissues. Proc Natl Acad Sci USA.

[73] Ni P, Ma X, Lin Y, Lao G, Hao X, Guan L (2015). Methionine sulfoxide reductase A (MsrA) associated with bipolar I disorder and executive functions in A Han Chinese population. J Affect Disord.

